# Tactile C fibers and their contributions to pleasant sensations and to tactile allodynia

**DOI:** 10.3389/fnbeh.2014.00037

**Published:** 2014-03-06

**Authors:** Jaquette Liljencrantz, Håkan Olausson

**Affiliations:** ^1^Institute of Neuroscience and Physiology, Gothenburg UniversityGothenburg, Sweden; ^2^Department of Clinical Neurophysiology, Sahlgrenska University HospitalGothenburg, Sweden

**Keywords:** touch, unmyelinated, tactile allodynia, fMRI, psychophysics, social

## Abstract

In humans converging evidence indicates that affective aspects of touch are signaled by low threshold mechanoreceptive C tactile (CT) afferents. Analyses of electrophysiological recordings, psychophysical studies in denervated subjects, and functional brain imaging, all indicate that CT primary afferents contribute to pleasant touch and provide an important sensory underpinning of social behavior. Considering both these pleasant and social aspects of gentle skin-to-skin contact, we have put forward a framework within which to consider CT afferent coding properties and pathways—the CT affective touch hypothesis. Recent evidence from studies in mice suggests that CTs, when activated, may have analgesic or anxiolytic effects. However, in neuropathic pain conditions, light touch can elicit unpleasant sensations, so called tactile allodynia. In humans, tactile allodynia is associated with reduced CT mediated hedonic touch processing suggesting loss of the normally analgesic effect of CT signaling. We thus propose that the contribution of CT afferents to tactile allodynia is mainly through a loss of their normally pain inhibiting role.

Historically, human tactile sensibility was considered to be mediated solely by low-threshold mechanoreceptors with large myelinated (Aβ) afferents conducting impulses at high speed (around 50 m s^−1^). In contrast, unmyelinated low-threshold mechanoreceptive afferents (C-LTMRs) have been known to exist in the hairy skin of mammals since 1939 (Zotterman, 1939; Douglas and Ritchie, 1957; Bessou et al., 1971; Iggo and Kornhuber, 1977; Kumazawa and Perl, 1977). For long, it was assumed that humans did not share this seemingly primitive tactile system with other mammals. Nevertheless, in recent years it has been demonstrated repeatedly that human skin is also innervated by C-LTMRs conducting impulses with a speed of only about 1 ms^−1^. In man, these nerve fibers were first found in microneurography recordings from the infra- and supra-orbital nerves (Johansson et al., 1988; Nordin, 1990). Soon after, they were found in the arm and leg suggesting a more general distribution (Vallbo et al., 1993, 1999; Edin, 2001; Wessberg et al., 2003; Campero et al., 2011). In humans, C-LTMRs are called C tactile or CT afferents but so far afferent response properties seem to be similar across species (Vallbo et al., 1999).

Although there is currently no accurate method to assess the innervation density of CT afferents in humans, it is a recurring experience in microneurography recordings from the lateral antebrachial cutaneous nerve of the forearm that they are encountered as often as Aβ afferents. CT afferents have never been found in the palm of the hand despite numerous microneurography recordings from this skin area.

## C tactile (CT) afferents

**Figure 1:**
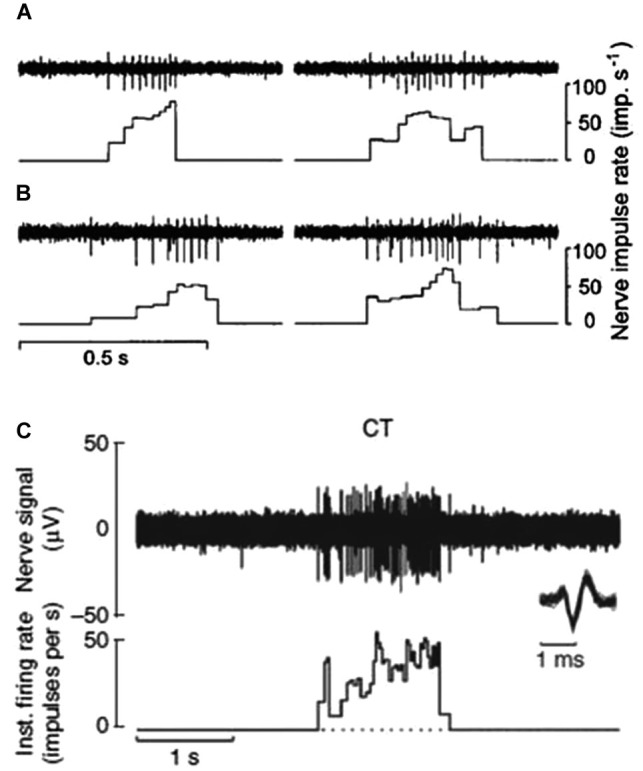
**CT afferent response to slowly moving mechanical stimulation.** **(A** and **B)** Responses of two separate units to the experimenter stroking his finger tip over the receptive field. Peak impulse rates were 78 and 64 imp/s **(A)** and 52 and 73 imp/s **(B)** (Vallbo et al., 1999). **(C)** Microneurography recording of nerve response and interspike interval histogram for another single CT afferent to soft brush stroking with a velocity of 3 cm s^-1^ and calibrated normal force of 0.4 N. Individual nerve spikes are superimposed on an expanded time scale below the nerve recording to illustrate the typical C impulse shape with a prominent negative peak (Loken et al., 2009).

CT afferents respond to indentation forces in the range 0.3–2.5 mN (Vallbo et al., 1999), tested with von Frey monofilaments, and are thus as sensitive to skin deformation as many of the Aβ afferents. CT afferents respond with high frequency to stimuli that are clearly innocuous, such as slow stroking with the experimenter’s finger tips or a soft brush (Figures 1A–C; Vallbo et al., 1999). In contrast to C nociceptors, with mechanical thresholds >2.5 mN, CT afferents do not distinguish between pin pricks and smooth probe indentations but respond equally well to both these types of stimuli. C nociceptors may also respond to light brush stroking but their responses never exceed a few impulses (Vallbo et al., 1999).

The conduction velocity of CT afferents, as assessed with mechanical or electrical stimulation, varies between 0.6–1.3 ms^−1^. To a sustained indentation, CTs initially respond with a high frequency burst of impulses but the firing rate decreases to zero within 5 s. The adaptation characteristic of CT afferents is thus intermediate in comparison with the slowly and rapidly adapting myelinated mechanoreceptors; slowly adapting units continue to fire during indentation whereas rapidly adapting units only fire when the skin deformation is changing. In a subset of CT afferents the response may increase again after the initial period of adaptation with firing continuing for 1–2 min until it finally stops; a phenomenon described as delayed acceleration (Vallbo et al., 1999). A related phenomenon has been described for rat nociceptors (Andrew and Greenspan, 1999). Another feature of CT afferents is that they are highly fatigable. When several identical stimuli are delivered to the same skin area the response to the first stimulus is usually much larger than the following responses to identical stimuli. When a skin deformation is released CT afferents may produce after-discharges that may last up to several seconds (Nordin, 1990).

**Figure 2:**
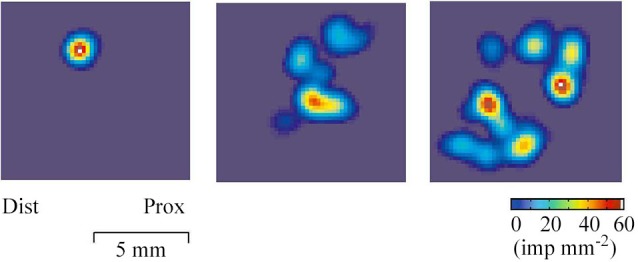
**Field geography of CT afferents on the forearm skin**. Color coded two-dimensional density plots of receptive fields of three CT afferents. The colors represent intensity of afferent firing. The geography of receptive fields was explored with a robotic scanning method: a lightweight probe with a small and rounded tip was made to scan the field area in a series of closely adjacent tracks while single unit activity was recorded (Wessberg et al., 2003).

The receptive field of a human CT afferent is roughly round or oval in shape with no preferred orientation. Detailed analyses has revealed that, in humans, the field consists of 1–9 small responsive hot spots distributed over an area up to 35 mm^2^ (Figure 2; Wessberg et al., 2003).

**Figure 3:**
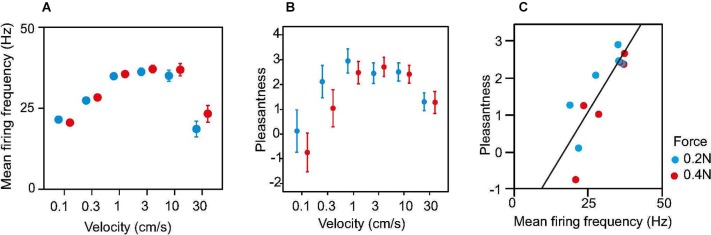
**Neural discharge rate and perception of pleasantness in response to soft brush stroking**. **(A)** Dots show average discharge rates during brush stroking for 16 CT afferents. **(B)** Average ratings of perceived pleasantness in response to soft brush stroking. Data are from 10 subjects. **(C)** Ratings of pleasantness as a function of neural discharge rate in CT afferents. Mean pleasantness ratings are plotted against the corresponding mean firing frequency for each brushing velocity and force. The plot is based on the data in **A** and **B**. The linear correlation was significant (Pearson’s linear regression, *R*^2^ = 0.70, *P* = 0.00063). Error bars show s.e.m.

A caressing type of slowly moving touch is a particularly effective stimulus for CT afferents. It has been measured through single unit microneurography that the maximal unit response occurs for movement velocities in the range 1–10 cm s^−1^ whereas the response is weaker for slower and faster movements (Loken et al., 2009; Figure 3A). In psychophysical experiments brush stroking in the same velocity range 1–10 cm s^-1^ is perceived as more pleasant than stroking with slower or faster velocities (Figure 3B). Indeed, there is a positive correlation between firing frequency of CT afferents and perceived pleasantness of soft brush stroking (Figure 3C).

## Findings in subjects lacking large myelinated (Aβ) afferents

Direct evidence for a specific role of CT afferents in tactile sensation has been difficult to acquire; a major reason being that it is not possible to stimulate CT afferents without also activating Aβ afferents. Unique data has been collected from two subjects selectively lacking Aβ afferents but who have intact C fibers as the result of sensory neuronopathy (a rare disorder of nerve cell bodies of the large primary sensory neurons) (Sterman et al., 1980). The two subjects (initials GL and IW) are well described in the literature (Forget and Lamarre, 1987; Cole and Sedgwick, 1992). They have been studied extensively over the years particularly with regard to motor functions because of their proprioceptive deficit. It had also been reported, although merely in passing, that they had lost all tactile sensations when they became ill. This observation was consistent with the view at that time that tactile sensation was altogether dependent on Aβ afferents. The dependency on Aβ signaling was largely based on nerve block experiments in healthy subjects demonstrating a lack of tactile sensations when Aβ fibers were blocked through pressure applied on the nerve (Mackenzie et al., 1975).

When it became evident that human skin is supplied with a system of unmyelinated afferents, it became necessary to re-examine the tactile sensibility of these rare neuronopathy subjects using more refined approaches. Rigorous psychophysical tests were pursued to explore if the neuronopathy subjects were able to detect CT targeted touch. It was found that subjects lacking Aβ afferents detected soft brush stroking and weak monofilament indentation on the forearm skin where CT afferents are abundant (Olausson et al., 2002, 2008; Cole et al., 2006). Importantly, they failed altogether to detect the same kind of stimuli applied to the glabrous skin of the hand where CT afferents are lacking. In addition, they were unable to detect vibratory stimuli which give a poor activation of CT afferents but a vigorous activation of Aβ afferents (Iggo, 1960; Bessou et al., 1971; Kumazawa and Perl, 1977; Olausson et al., 2002, 2008).

The sensation reported by the patients in association with massive and selective CT input (soft brush stroking of the hairy skin) was weak, vague, and inconsistent. In some trials the subject reported no sensations at all. In others, they reported a sensation of light touch which was barely detectable and difficult to describe. One of the subjects (GL) reported that she began to feel more touch sensations in her daily life once she had had the experience of touch perception from the affected skin areas during the experiments and had become aware of this type of perceptual experience. Although the two neuronopathy subjects were not able to give a concise or detailed description of the sensation elicited by CT stimulation, they both reported, independent of each other, that it was a pleasant touch experience with no hint of pain, tickle, or itch. None of the two neuronopathy subjects feel tickle in the affected skin areas which contradicts the old hypothesis that CTs may signal a tickling sensation (Zotterman, 1939; but see Fukuoka et al., 2013). The neuronopathy subjects’ ability to spatially localize CT stimulation is very poor; they make mistakes when trying to identify which body quadrant is being stimulated, although they overall perform above chance level (Olausson et al., 2008).

## Findings in subjects lacking C afferents

We have also examined patients with a hereditary disorder associated with a nerve growth factor beta (NGFB) gene mutation causing a denervation pattern opposite to that of the neuronopathy subjects GL and IW. Carriers of the NGFB mutation show a reduction in density of thinly myelinated and unmyelinated nerve fibers, thus likely including CT afferents, whereas their Aβ afferents are intact. Their condition has been classified as hereditary sensory and autonomic neuropathy type V (HSAN-V). We have addressed the relationship between C fiber function and pleasant touch perception in 10 HSAN-V individuals from a unique population of carriers (Morrison et al., 2011). The HSAN-V patients perceive gentle, slow stroking, optimal for eliciting CT afferent responses (1–10 cm s^−1^), as less pleasant than do matched controls and also differ in their rating patterns across stimulation velocities. Hence, these observations further support the notion that CT afferents make a critical contribution to the perception of affective touch. 

## Cortical processing of C tactile (CT) stimulation

**Figure 4:**
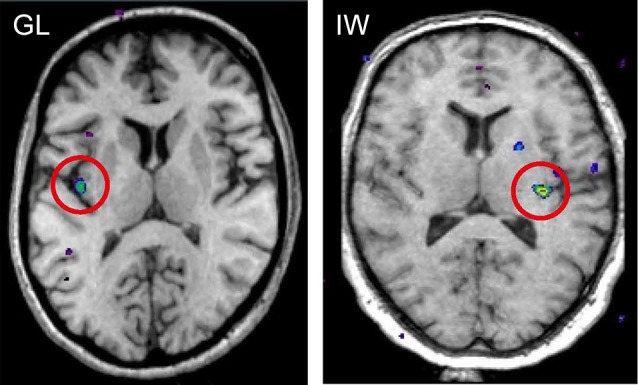
**fMRI activation in posterior insular cortex evoked by selective stimulation of CT afferents in the neuronopathy subjects GL and IW lacking Aβ afferents**. In both subjects, the posterior insular activation was contralateral to the stimulated forearm and reflects differences in blood oxygen level dependent (BOLD) signal during soft brush stroking and a baseline condition of rest (Olausson et al., 2002; Olausson et al., 2008).

When functional magnetic resonance imaging (fMRI) is used to study brain responses to touch stimuli in neurologically intact subjects and in neuronopathy subjects lacking Aβ afferents, different sensory areas are activated by Aβ and CT afferents. In healthy subjects soft brush stroking activates the classical somatosensory areas S1 and S2 as well as insular cortex, notably the posterior part of the contralateral insular cortex (Olausson et al., 2002). When similar brushing stimuli are applied to the neuronopathy subjects lacking Aβ afferents (GL and IW) only the posterior insular region is activated (Olausson et al., 2002, 2008; Figure 4). Further, there is a somatotopic organization of CT responses in the posterior insular cortex with forearm projecting anterior to thigh stimulation (Bjornsdotter et al., 2009). The somatotopic arrangement suggests that CT afferents follow the thin-fiber spinothalamic pathway with the posterior insular cortex as the primary cortical receiving area (Craig, 2002). No corresponding insular activation was found for brush stroking in the C-fiber denervated HSAN-V subjects (Morrison et al., 2011).

CTs have not been found in the glabrous skin of the hand, yet it is commonly observed that glabrous skin touch is also perceived as pleasant. When contrasting the brain activation of slow brush stroking on the forearm to that of slow brush stroking in the palm there is a significantly greater activation of the posterior insular cortex and mid-anterior orbitofrontal cortex (OFC) for brush stroking on the hairy skin of the forearm (McGlone et al., 2012). The opposite contrast (stroking on the arm minus stroking in the palm) shows a significant activation of somatosensory cortices. Although psychophysical ratings show no differences in intensity or pleasantness ratings, a touch-questionnaire in which subjects used a newly developed “Touch Perception Task” (Guest et al., 2011) shows a significant difference for the two body sites; emotional descriptors are rated higher on the forearm and sensory discriminatory descriptors are rated higher on the palm (McGlone et al., 2012). These findings are consistent with the hypothesis that CT targeted touch from hairy skin is processed in limbic cortical areas and represents an innate non-learned process. In contrast, pleasant touch from glabrous skin, mediated by Aβ afferents, is processed in somatosensory cortex and represents an analytical process dependent on previous tactile experiences (McGlone et al., 2012).

In addition to the insular cortex and the OFC, the posterior superior temporal sulcus and the medial prefrontal cortex/dorsoanterior cingulate cortex have also been implicated in processing CT targeted touch (Lindgren et al., 2012; Bennett et al., 2013; Gordon et al., 2013; Voos et al., 2013).

## The C tactile (CT) affective touch hypothesis

Microneurography recordings indicate that CT processing is tuned to the slow, dynamic properties of a light touch on hairy skin (Loken et al., 2009). Strikingly, these aspects of touch tend to be salient in affiliative tactile interactions between individuals (Gallace and Spence, 2010). Building on the intriguing similarity between socially-relevant touch and the class of preferred stimuli for CT activation, we have thus proposed a CT- affective (or social) touch hypothesis that seeks to account for the known properties of CT afferents, their central projection and perceptual impact (Vallbo et al., 1999; Morrison et al., 2010).

Affective touch may constitute a distinct domain of touch, characterized not by its sensory-discriminative functions, but by its social context and accompanying subjective component. As such, social touch may draw on a functionally and qualitatively different kind of information than that coded by Aβ afferents, requiring specialized functional organization in both the periphery and the central nervous system. CT afferents may thus constitute a privileged peripheral pathway for tactile stimulation that is likely to signal close, affiliative body contact with others (Morrison et al., 2010).

## C tactile (CT) afferents and tactile allodynia

Tactile allodynia is a symptom of neuropathic pain where normally innocuous moving tactile stimuli produce pain. People with tactile allodynia typically experience a burning, tender sensation during soft stroking of the affected skin (Rasmussen et al., 2004). Even a very light stimulus, such as a patient’s garment brushing against the skin during movement, can evoke allodynia. The prevailing hypothesis for tactile allodynia is changed tactile signaling in the spinal cord (Woolf, 1993) following central sensitization where Aβ LTMRs signal to nociceptive neurons in the dorsal horn and from there to cerebral pain processing areas (Campbell et al., 1988; Torebjork et al., 1992; Woolf, 1993; Wasner et al., 1999). This view is based on human selective nerve block experiments demonstrating that tactile allodynia is abolished by compression or ischemic block of Aβ afferents (Gracely et al., 1992; Torebjork et al., 1992; for contradictory results see Nagi et al., 2011).

The view of a critical role for Aβ afferents in mediating human tactile allodynia was established at a time when C-LTMRs were generally thought not to exist in humans. The first study to suggest a critical role for C-LTMRs in signaling allodynia used a vesicular glutamate transporter type 3 (VGLUT3) knock-out mouse, which functionally disconnects signaling from C-LTMRs by preventing glutamate release (Seal et al., 2009). After the loss of VGLUT3 neurons mechanical hypersensitivity following inflammation, nerve injury and trauma is reduced, thus suggesting a critical role for C-LTMRs in mechanical hypersensitivity (Seal et al., 2009). Furthermore, electrophysiological recordings in rats demonstrate a possible anatomical pathway for tactile allodynia where C-LTMRs project to lamina I spinoparabrachial wide dynamic range neurons (Andrew, 2010). Nevertheless, a later study found that preventing the development of C-LTMRs resulted in mice with no hypersensitivity (Lou et al., 2013).

Recently, new light has been shone on this question through the identification of the C-LTMR specific marker TAFA4 (Delfini et al., 2013). Following inflammation and nerve injury TAFA4 knock-out mice show enhanced mechanical and chemical hypersensitivity, and this effect is reversed by application of the TAFA4 protein (Delfini et al., 2013). The authors speculate that upon activation, C-LTMRs might release both glutamate and TAFA4 with glutamate promoting mechanical hypersensitivity and TAFA4 instead preventing mechanical hypersensitivity. This suggestion also provides a potential explanation for the different findings regarding the functional knock-out of glutamate signaling (Seal et al., 2009) and the complete loss of C-LTMRs (Lou et al., 2013). Losing glutamate alone as in the study by Seal et al. would leave TAFA4 unopposed and drive resistance to hypersensitivity. However, in the case of a complete loss of C-LTMRs (Lou et al., 2013) both glutamate and TAFA4 would be lost leaving no net change in sensitivity. But there are other potential explanations for the discrepancy between these two studies. For example, in Lou et al. experiments there is a disrupted development, so the results might reflect compensation for growing up without C-LTMRs. Alternatively, spinal cord and brain neurons, which also express VGLUT3, may mediate the injury-induced hypersensitivity seen in Seal et al. experiments, rather than C-LTMRs.

A pain modulatory role for C-LTMRs was suggested earlier in a study in rats indicating that C-LTMR targeted input may inhibit C-nociceptive messages in the dorsal horn (Lu and Perl, 2003). By conducting electrophysiological experiments, a specific inhibitory pathway was identified between substantia gelatinosa neurons receiving direct peripheral C-LTMR afferent projections and other substantia gelatinosa cells receiving direct nociceptive input (Lu and Perl, 2003). This unmyelinated circuit represents a potential pathway for C-LTMR impulses to suppress nociceptive impulses (Lu and Perl, 2003). Further, in wild-type mice administration of TAFA4 reverses the effect of injecting an inflammatory agent (carrageenan) normally causing mechanical hypersensitivity, consistent with an analgesic role for C-LTMRs (Delfini et al., 2013).

The topic of C-LTMRs in pain inhibition also ties back to the finding of pharmacogenetic activation of MRGPRB4^+^ expressing neurons (thought to be C-LTMRs) promoting conditioned place preference in mice, indicating that such activation is positively reinforcing and/or anxiolytic (Vrontou et al., 2013), mechanisms which also may have a role in pain modulation.

Based on this animal literature we set out to examine the contribution of CT afferents to the allodynic condition in humans using the heat capsaicin model of dynamic tactile allodynia (Liljencrantz et al., 2013). The contribution of CT afferent signaling was addressed by studying healthy subjects as well as the two rare patients with selective denervation of Aβ afferents (GL and IW). Following application of the model healthy subjects reported tactile evoked pain whereas the patients did not. Instead, both subjects spontaneously reported that the stroking sensation from the allodynic zone was different to their C-touch sensation (faint sensation of pleasant touch) familiar to both subjects. When asked to further describe how the sensation differed, they both, independent of each other, said “weaker sensation” for stimuli in the allodynic zone. These subjective differences between the allodynic and control zones were quantified and found to be significant in forced-choice testing (Liljencrantz et al., 2013). fMRI in healthy subjects and in one scanned patient (GL) indicates that stroking in the allodynic and control zones evoke different responses in the posterior insular cortex. In addition, there is a reduced activation in the OFC for stroking in the allodynic zone compared to a control area. Since the OFC is a key area for CT hedonic processing (McGlone et al., 2012; cf. above) these findings suggest that dynamic tactile allodynia is associated with reduced CT mediated hedonic touch processing. Nevertheless, since the patients do not develop allodynic pain (Treede and Cole, 1993; Liljencrantz et al., 2013), this seems dependent on Aβ signaling, at least under these experimental conditions.

Considering a possible analgesic effect of C-LTMR signaling (Lu and Perl, 2003; Delfini et al., 2013; Vrontou et al., 2013) it seems pertinent to speculate that in neuropathic pain conditions there is a gating resulting in a loss of the pain inhibition mediated by C-LTMRs to prioritize nociceptive signaling. This would be consistent with the canonical view that tactile allodynia is signaled by Aβ afferents, and we thus propose that the contribution of C-LTMR/CT afferents is mainly through a loss of their normally pain inhibiting role.

## Conflict of interest statement

The authors declare that the research was conducted in the absence of any commercial or financial relationships that could be construed as a potential conflict of interest.
